# Development and Validation of the Short Healthy Eating Index Survey with a College Population to Assess Dietary Quality and Intake

**DOI:** 10.3390/nu12092611

**Published:** 2020-08-27

**Authors:** Sarah Colby, Wenjun Zhou, Chelsea Allison, Anne E. Mathews, Melissa D. Olfert, Jesse Stabile Morrell, Carol Byrd-Bredbenner, Geoffrey Greene, Onikia Brown, Kendra Kattelmann, Karla Shelnutt

**Affiliations:** 1 Department of Nutrition, University of Tennessee, Knoxville, TN 37996, USA; clessard@vols.utk.edu; 2 Department of Business Analytics and Statistics, University of Tennessee, Knoxville, TN 37996, USA; wzhou4@utk.edu; 3 Department of Food Science and Human Nutrition, University of Florida, Gainesville, FL 32611, USA; anne.mathews@ufl.edu; 4 Department of Human Nutrition and Foods, West Virginia University, Morgantown, WV 26506, USA; Melissa.Olfert@mail.wvu.edu; 5 Department of Agriculture, Nutrition, and Food Systems, University of New Hampshire, Durham, NH 03824, USA; jesse.morrell@unh.edu; 6 Department of Nutritional Sciences, Rutgers University, New Brunswick, NJ 08901-8554, USA; bredbenner@sebs.rutgers.edu; 7 Department of Nutrition and Food Sciences, University of Rhode Island, Kingston, RI 02881, USA; ggreene@uri.edu; 8 Department of Nutrition, Dietetics, and Hospitality Management, Auburn University, Auburn, AL 36849, USA; onb0001@auburn.edu; 9 Department of Health and Nutritional Sciences, South Dakota State University, Brookings, SD 57007, USA; Kendra.Kattelmann@sdstate.edu; 10 Department of Family, Youth, and Community Sciences, University of Florida, Gainesville, FL 32611, USA; kpagan@ufl.edu

**Keywords:** the HEI, survey, diet quality

## Abstract

Because diet quality (DQ) is associated with risk of chronic disease and is a common construct assessed in health-related research, validated tools to assess DQ are needed that have low respondent and researcher burden. Thus, content experts develop the Short Healthy Eating Index (sHEI) tool and an associated scoring system. The sHEI scoring system was then refined using a classification and regression tree (CRT) algorithm methodology with an iterative feedback process with expert review and input. The sHEI scoring system was then validated using a concurrent criterion validation process that included the sHEI DQ scores (calculated from responses from 50 participants) being compared to the participants’ Healthy Eating Index scores derived from 24 h recalls. The total HEI score from the CRT algorithm highly correlated with the 24 h recall HEI score (0.79). For individual food group items, the correlation between the CRT algorithm scoring and the 24 h recall data scoring ranged from 0.44 for refined grains to 0.64 for whole fruits. The sHEI appears to be a valid tool for estimating overall dietary quality and individual items (with correlations > 0.49) for fruits, vegetables, dairy, added sugar, sugar from sugar-sweetened beverages, and calcium.

## 1. Introduction

The impact of what an individual eats on health outcomes is rarely influenced by any one eating event or single food [1]. Instead, health outcomes related to diet are a result of complex combinations of foods eaten together over time, which is referred to as dietary quality (DQ) [1,2]. Low DQ, such as excessive sugar intake or not meeting guidelines for fruit and vegetable consumption, has been associated with increased risk of obesity and other chronic diseases [3,4]. Alternatively, high DQ has been suggested to reduce the risk and be a protective factor against chronic diseases, such as Type 2 Diabetes Mellitus (DM), cardiovascular disease (CVD), and some cancers [5,6]. A review conducted by Wirt and colleagues found that higher DQ was inversely associated with chronic disease risk and served as a protective factor against adverse health outcomes such as all-cause mortality, CVD mortality, CVD risk, cancer mortality, and all-cancer risk [2]. Another study found that for every additional serving of fruit and vegetable, stroke risk decreased by 11% and 3%, respectively [7]. Diets high in dietary fiber may reduce the risk of some cancers, such as colorectal and breast cancer [8,9]. Additionally, greater intake of leafy greens was associated with a 14% reduced risk of DM [10], and leafy greens and berry intake has been associated with a decreased risk of cognitive decline or developing dementia [11]. Since DQ is associated with many chronic disease outcomes, DQ is an important outcome to assess in nutrition, health, and chronic disease prevention research and program evaluation [12]; however, measuring DQ can be a complex process.

There are a various tools to measure DQ, including: the Diet Quality Index-Revised (DQI-R), the DQI-International (DQI-I), the Mediterranean Diet Score (MDS), the Mediterranean Diet Quality Index (KIDMED), the MIND diet score, and the Healthy Eating Index (HEI). The DQI-R, which was revised in 1999 to reflect new US food and nutrition guidelines, provides DQ indicators, including assessing moderation, variety, and proportionality in the diet [13]. The index includes 10 components, each worth 10 points, to provide a total score of 100 [13]. Though the DQI-R does not correlate well with energy or protein intake, it does measure calcium and iron intake as separate components [13]. The DQI-I was developed and validated with international populations from existing DQ scores such as the DQI-R and the HEI [14]. This tool has the ability to compare dietary qualities across different cultures and different dietary patterns [14]. The DQI-I includes four components in the overall index score: diversity, adequacy, moderation, and balance. The DQI-I has the ability to measure nutrition transition to determine changes in food patterns in cultures across time, but some cultural foods may not be in the nutrient databases, which may underestimate some food groups such as fiber [14].

The KIDMED provides a DQ score for Mediterranean diet patterns in children and adolescents, as well as college students [15,16]. Scores range from 0 to 12, with a score of greater than 8 indicating high adherence to the Mediterranean diet. Although the index provides an overall score of adherence to the diet for individuals aged 2–24 years, it is not generalizable to cultures that may not have access to or follow this eating pattern. Additionally, KIDMED does not provide component scores as seen in some other DQ measures [13,14,15]. Another method that assesses components of the Mediterranean diet is the MIND (Mediterranean–DASH Intervention for Neurodegenerative Delay) tool [11]. The MIND tool collects dietary information on food components that are associated with positive health outcomes and have been found to be neuroprotective [11]. The MIND score is calculated using a semiquantitative Food Frequency Questionnaire (FFQ) of foods found in the Mediterranean diet, and identifies 10 brain healthy food groups, and five unhealthy food groups [11]. Each component is assigned a score and the total sum provides the MIND diet score [11].

A review by Burggraf and colleagues assessed the construction process of 57 different indices for DQ that have been developed across the globe [17]. Each index has varying criteria such as theoretical framework (e.g., dietary recommendations, diet pattern, and population), index dimensions and structure, indicator selection, normalization methods (e.g., how dietary variables with different units are standardized and how cutoff values are identified to normalize data), and aggregation technique (e.g., how DQ components are combined to make a composite score) [17]. Several measures have been validated for the US population, such as the Dietary Quality Index-Revised [13], Dietary Behavior Score-2009 [18], and the Modified Mediterranean Diet Score-2014 [19]; however, the HEI-2015 has been recommended as the most appropriate measure of DQ for the US population based on the preferred construction method cited in the dietary quality indices review [17]. Another review that assessed how well DQ indices could predict the risk of obesity, found that the HEI was the best DQ index predictor of obesity risk over others [20]. Another study found that the HEI scoring method meets the criteria to effectively follow dietary guidelines, thus supporting the use of the HEI to determine DQ [21].

The HEI score indicates how well diets compare to the Dietary Guidelines for Americans with scores ranging between 0 and 100 (higher scores indicate higher DQ) for Americans aged 2 years and up [22,23]. The HEI was developed in 1995 to understand how closely American diets match national recommendations [22]. A major revision in the scoring tool was completed in 2005, and the most recent update of the HEI was in 2015 to reflect the 2015–2020 Dietary Guidelines for Americans [24,25,26]. The scoring system includes nine components that are encouraged for a healthful diet and four components that should be consumed in moderate amounts to maintain a healthful diet [24]. Multiple methods were used to evaluate each version of the HEI, such as assessing content validity, construct validity, and reliability [24,25,26]. The HEI is validated using data from the National Health and Nutrition Examination Survey (NHANES) [24,25,26]. Strengths of the HEI score include providing high scores for diets known to be high in quality, being sensitive enough to show differences in scores between individuals with different known diets, and providing multiple dimensionality of diet [24]. However, like all assessment tools that rely on self-reported measures, the HEI is subject to measurement error [24]. In addition, since the total HEI score does not highlight individual components of diets or quantities of foods consumed, it is possible that a single or a few food component(s) could be inaccurately reported or excessively consumed and unduly influence the total DQ score. Because of this potential influence of a single or few food item(s), investigations of each individual food component should be considered when interpreting the total DQ score [24]. High DQ as scored by the HEI also has been found to not be strongly predictive of health outcomes [2,21]. The HEI has many different purposes including epidemiological research, population health tracking, and evaluations of nutrition assistance programs, food environments, and diet-related interventions; however, shortcomings of the HEI may make it inappropriate for assessment of the DQ for individual diets [25].

There are no specific instruments designed to be administered to individuals, scored and then used to produce a HEI score. Instead, DQ must be calculated by using the tools with food consumption assessments such as from food records or 24 h recalls (24 h recalls). Thus, identifying foods consumed is a critical first step in the process of determining DQ. Foods consumed can be assessed using food records, 24 h recalls, or Food Frequency Questionnaires (FFQs) [12]. Although these are considered valid methods to assess dietary intake, each method has factors that limit their usefulness [12]. Food records (which collect food and beverages consumed, usually for a set amount of time) are subject to respondent burden and researcher burden (specialized training, time-consuming data entry, and costly software) [12]. Though food records do not rely on the participant’s memory (because participants are logging intake in real time), detailed training and commitment is required for participants to fill out food records accurately and completely [12]. In addition, the information collected may not accurately reflect the average DQ or usual individual food group intake patterns of an individual because of issues of reactivity bias, when participants change their diet to make recording easier or to appear to eat more healthfully or from seasonal variations [12].

Requiring less respondent burden, but comparable researcher burden, 24 h recalls can be used to determine DQ using the HEI as well as identifying individual food group intake patterns [12]. Drawbacks related to 24h recalls include within-person random error, memory deficits, inaccurate reporting due to social desirability bias, need for repeated administrations, respondent memory deficits, cost of associated data software needed for analysis, intensive time requirements for data entry, and analysis needed from trained researchers [12].

Requiring the least amount of respondent and researcher burden, FFQs can be used to capture food and beverage consumption over a certain period of time (e.g., the last week or month) and are designed to measure either the entire diet or specific portions of a diet. Commonly used validated FFQs include the Harvard Food Frequency Questionnaire, Diet History Questionnaire [27], Block Food Frequency Questionnaire [28], and NCI Diet History Questionnaire [29]. Although less respondent burden than food records or 24 h recalls, most FFQs still often take 30–60 min to complete. If data are collected on paper forms, data entry can also be time consuming for researchers and subject to data entry error (as the responses need to be entered by trained researchers into a software program). Although some FFQs can be used to estimate DQ, one major limitation is the accuracy of FFQs to estimate energy whereas energy intake can be calculated more accurately from either 24 h recalls or food records [12]. Another limitation of FFQs is that less specific and detailed dietary information is captured by FFQs than from either 24 h recalls or food records. Dietary screeners, similar in format to FFQs, can provide information on individual food group consumption, but cannot be used to estimate DQ [12].

All the methods of dietary intake assessment previously described (food records, 24 h recalls, FFQs, and dietary screeners) are subject to inaccuracy because they rely on self-reported dietary information and are subject to both over and underreporting errors [12,30,31,32]. Screeners vary in length and included components, and many are calibrated to 24 h recall data to produce better predicted outcomes [33]. It is recommended for all self-reported data, whether it be food record, 24 h recall, FFQ, or screener to accompany biomarker data to ensure higher accuracy of dietary intake [12].

Biomarker assessments can precisely detect nutrient levels through blood and urine tests. Biomarkers that can be used to measure different components of dietary intake include carotenoids, fatty acids, vitamins, polyphenols, food contaminants, inorganic compounds, and enzymes [34]. Though biomarkers are not subject to inaccuracies related to self-report data, they can be invasive, costly, inappropriate for research conducted remotely, nutrient specific, and many are still in development; therefore, are not widely accessible [35]. There is also currently no single (or specific combination) biochemical assessment that provides an overall picture of DQ [36]. However, dermal carotenoid levels, assessed via serum analysis or resonance Raman spectroscopy (RRS: a small laser with blue wavelength applied to one finger), have been found to be correlated with fruit and vegetable intake [37,38], and fruit and vegetable intake has been found to be correlated with the overall healthfulness of the diet [7,10,23]. Carotenoid levels measured via RRS provide a non-invasive, quick, and objective measurement of overall DQ [39]; however, the instrumentation required is costly and may not be accessible to many research and program evaluation studies.

There are also other advancements being made in dietary assessment technology that use image assistance on wearable cameras or mobile phones that can assess DQ [40,41]. These methods are thought to be more precise because they reduce underreporting rates and are more accepted by participants [41]. However, one study found that image assisted dietary assessment still had a 12% prevalence of underreporting in men and 10% prevalence in women, suggesting this technology may be comparable to other self-reported methods [42]. Regardless, most of these technologies still require more development, are costly, and are not readily accessible [40,41].

Unless new objective methods become widely available and accessible that better assess DQ, assessment tools for DQ are needed that require less respondent and researcher burden. The purpose of this study was to develop and validate a short tool that could be used to assess DQ and individual food groups consumed while reducing the respondent and researcher burden associated with current available dietary assessment methodologies. Survey tools can be developed and validated in many ways, and there is no one right way [43]. However, validating a new tool against multiple previously validated tools that are used to assess the same constructs increases the validation of the new tool being developed [44]. To achieve this purpose, the following two objectives were pursued:

1. Determine whether the Short Healthy Eating Index (sHEI) could be used to accurately estimate dietary intake of food groups by comparing the newly developed sHEI survey outputs to those of the Dietary Screener Questionnaires (DSQs) [33], 24 h recalls, and carotenoid measurements.
2. Determine whether the sHEI could be used to accurately estimate the HEI score calculated from 24 h recall data.

## 2. Materials and Methods

**Figure 1:**
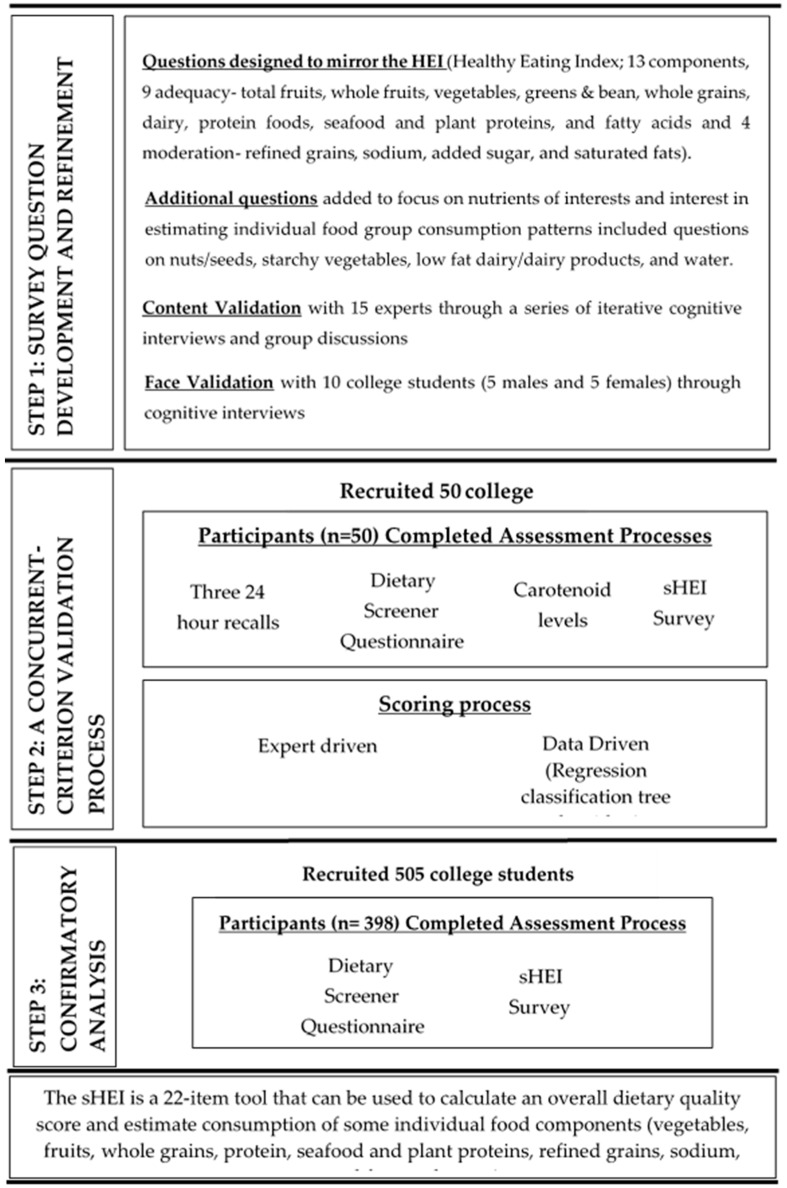
Process of developing and validating a survey tool (the sHEI) to estimate dietary quality and individual food group consumption of vegetables, fruits, whole grains, protein, seafood and plant proteins, refined grains, sodium, saturated fats, and water.

A sHEI was developed and validated on one university campus as a part of a larger project focused on promoting health on college campuses. Because improved dietary behavior was a primary outcome of the larger project, a validated and short tool was needed to assess dietary behaviors of college students. The survey development and validation process followed a three-step process (Figure 1). In step one, survey questions were developed and refined. In step two, a concurrent criterion validation process was used to determine whether a DQ score calculated using the newly developed sHEI correlated with three existing validated measurements (the HEI calculated from repeated 24 h recalls, the DSQ, and dermal carotenoid levels). In step three, a confirmatory analysis process was conducted to confirm the correlation of the sHEI and the DSQ with a larger sample. All methods were reviewed and approved by the Institutional Review Board at the University of Tennessee (UTK IRB-14-09366 B-XP). All data processing, scoring, and statistical analysis were done in R (Windows Version 4.0.0).

### 2.1. Survey Question Development and Refinement

A group of 15 content experts developed the initial survey questions through a process of repeated individual cognitive interviews and focus groups. Survey questions were developed to mirror the components of the HEI-2015 scoring system and allow for comparable scoring methodology. Interviews and focus groups continued until no further changes were recommended.

Cognitive interviews were then used for face validation of the survey using a response process approach with a convenience sample of 10 college students (5 males and 5 females) [45]. After completing the consent process, the participants completed the survey and then were interviewed by a trained researcher. The interview consisted of the participant reporting back what they thought the questions were asking, why they answered the way they did, and if they thought any changes were needed to the wording of the questions.

### 2.2. A Concurrent Criterion Validation Process

A concurrent criterion validation process was then used to determine whether the sHEI (35 items) accurately identified participants’ DQ and individual food group intake by comparing the developed tool to three existing validated measurements (the HEI calculated from repeated 24 h recalls, the DSQ, and dermal carotenoid levels).

Power analysis indicated that a sample of 50 participants would be adequate for determining the correlation of a newly developed tool with data collected via 24 h recalls; thus, 50 participants were recruited from the college campus via flyer, word of mouth, and class emails to participate in this validation project. Interested participants (who were students attending the university, 18 years of age or older, completed an online consent process, and who provided their contact preferences and information) were called between the hours of 11 a.m. and 6 p.m. (or other time indicated as preferred) to complete a 24 h recall by phone. Three recalls were completed on two non-consecutive weekdays and one weekend day. At the end of each recall, participants were asked whether the day reported on was a normal day (e.g., no special events/celebrations, trips taken, or illnesses). If they reported the day was unusual, another time was scheduled to repeat the recall, and the normal day recall was then used. All recalls were scheduled initially to be completed in a one-week process, but if a recall had to be rescheduled, the additional call could be completed in a second but consecutive week. All recalls were completed within a two-week period.

The recalls were conducted using the multiple-pass method documenting everything consumed (food and drink—including water) using the Nutrition Data System for Research (NDSR) software version 2017 developed by the Nutrition Coordinating Center (NCC), University of Minnesota, Minneapolis, MN, USA. At the conclusion of the third recall, a survey was sent via email to the participant. The survey included the sHEI as well as the DSQ, and demographic questions (date of birth, gender identity, race, and ethnicity). At the end of the survey, participants completed an online scheduling process to set an appointment for an in-person anthropometric assessment. The assessments were completed within a week of completing the survey and included a measurement of carotenoid levels. Single measurements of dermal carotenoid levels were collected twice using a carotenoid scanner (resonance Raman spectroscopy; RRS, Longevity Inc, Salt Lake City, UT, USA), which used a small laser to measure dermal carotenoid levels by placing a finger on the scanner [38]. An average of the two single measurements were used for analysis. All measurements were taken by trained researchers. Participants were provided a $50 gift card for participating in this study.

The food group consumption estimates from the sHEI were compared with 24 h recalls (using the NDSR output), the DSQ, and dermal carotenoid levels. The NDSR outputs for the HEI food component intakes were calculated using codes provided by the Nutrition Coordinating Center of the University of Minnesota [46]. All the food consumption variable (the sHEI, the NDSR, and the DSQ) values’ square roots were taken to create a more normal distribution. The NDSR output from this study’s three 24 h recalls were scored to produce consumption amounts of each HEI food group and the total HEI score [45]. The DSQ was scored using the current method from the National Cancer Institute [33]. This method uses regression models to predict food group consuption adjusting for age and gender. Descriptive statistics were used to describe demographics, lifestyle, and dietary patterns of participants using frequencies and relative frequencies for categorical variables and means and standard deviations for continuous variables.

To score the sHEI, two approaches were used. The first being a content expert-developed system, and the second being a data-driven approach. Content experts first transformed the HEI minimum consumption cutoff scoring to meet a 2000 calorie diet to be comparable to the servings reported by the sHEI. Because the HEI set a minimum amount of needed food group consumption to gain full points to the overall HEI score and the sHEI assessed how many servings per day were consumed (using photographs and written descriptions to help participants estimate the number of servings), content experts had to adjust the sHEI reports in servings per day to match what would meet criteria for receiving full points in the 2015 HEI. Therefore, the sHEI servings response options were transformed to consumption amounts (cup, ounce, teaspoon, or gram equivalents) that matched the NDSR units, and content experts identified calorie contributions for each sHEI serving size food group (e.g., 25 calories from ½ cup cooked vegetables, or 80 calories from ½ cup cooked pasta). Content experts identified the minimum consumption amounts required for each sHEI food group question needed to receive full points towards the total DQ score in sHEI analysis. All intake options below that minimum required for full points were then reduced in a stepwise approach. The scores were then reviewed for practical and clinical accuracy to identify any concerns.

The data-driven approach used a classification and regression tree (CRT) algorithm to extract rules for estimating the intake of each food group and the total energy consumed [47]. For each food group and the total DQ score variable, the true estimates from the NDSR were used as the dependent variable (i.e., target variable), and the sHEI variables as independent variables (e.g., input variable). The CRT algorithm automatically selected sHEI variables to split participants into groups and determine average consumption for each group. To allow for better generalizability, a minimum size of 20 was required for a node to consider splitting and the maximum depth of the tree was set to 5. Since the dependent variable was continuous (e.g., the food group intake), the CRT algorithm provided a path (automatically splitting responses into subnodes) seeking to reduce variance. The CRT algorithm was run using the Recursive Partitioning and Regression Trees (rpart) package (Version 4.1-15) in R.

The first 30 questions in the survey were transformed into 15 item questions for analysis by combining possible responses from two separate questions. There were 15 main food group questions with 15 follow-up questions that were only asked if the participant reported a non-daily intake of a particular food group. These two questions for each food group were coded into one ordinal variable with 11 possible levels: “never” (Level 1), “almost never” (Level 2), “a couple of times per year” (Level 3), “a couple of times per month” (Level 4), “a couple of times per week” (Level 5), “1 serving per day” (Level 6), “2 servings per day” (Level 7), “3 servings per day” (Level 8), “4 servings per day” (Level 9), “5 servings per day” (Level 10), and “6 or more servings per day” (Level 11). The last 5 questions in the sHEI were encoded as ordinal, each with 3 levels: “none/almost none” (Level 1), “some” (Level 2), and “a lot” (Level 3). Gender was assessed as a plausible input variable for all food groups to determine whether using the variable improved scoring accuracy; however, when gender was not picked up as a predictive independent variable in the CRT algorithm, gender was not included in the scoring rule procedure for that food group. This data-driven scoring process was then compared to the content expert scoring system to determine whether the scores generated were clinically equivalent, and they were found to be acceptable. The scores for the 13 HEI food components were then added to find the total sHEI score.

Pearson’s correlation coefficients were calculated to examine the strength of the sHEI estimates and the 24 h recalls, the DSQ, and dermal carotenoid level. Correlations between the sHEI and the total HEI scores were calculated; a correlation between the sHEI food groups and the DSQ food groups was calculated; and, a correlation between the sHEI green vegetables and the dermal carotenoid measurement was calculated. A coefficient with an absolute value less than 0.3 was considered negligible, between 0.3 and less than 0.5 was low, between 0.5 and 0.7 was moderate, and above 0.7 was considered highly correlated [48].

### 2.3. Confirmatory Analysis

To confirm that the developed sHEI scoring system produced an outcome equivalent to the DSQ, confirmatory analysis was completed with a convenience sample of college students. Participants were recruited via emailed and posted announcements sent by class instructors. After completing the online consent process, participants took an online survey that contained both the sHEI and the DSQ. Pearson’s correlation was used to compare and identify the level of agreement between the tools. Descriptive statistics were used to describe participant demographics.

## 3. Results

Of the fifty subjects recruited for the concurrent validation process, more than half were female (*n* = 30, 60%), most were non-Hispanic White (*n* = 41, 82%), age ranged from 18 to 35 years (with the mean and median both being 21 years), and BMI ranged from 17.9 to 49.1 (with mean 25.21 and standard deviation 5.28).

Table 1 summarizes food group consumption using the NDSR, the DSQ, and sHEI tools. Table 2 shows the correlations of the HEI component and total scores estimated by 24 h recall and the sHEI. The total sHEI score highly correlated with the 24 h recall score (0.79). For individual food group items, the correlation between the sHEI and the 24 h recall ranged from 0.44 for fatty acids (low correlation) to 0.64 for total fruits (moderate correlation).

### 3.1. DQ Scoring Rules Interpretation

Balancing the correlation performance and interpretability, the final scoring rules were selected from different possible approaches. Specifically, for total fruits, whole fruits, greens and beans, and added sugars, we adopted the expert-developed scoring rules; for total vegetables, whole grains, dairy, and seafood and plant proteins, we adopted rules derived with the CRT algorithm using select relevant sHEI items as predictors; and the remaining ones derived with the CRT algorithm using all sHEI items as the predictors. The last group using a purely data-driven approach was due to greatly enhanced performance whereas seeming unrelated items may be involved in the scoring rules. For example, green vegetables, seafood, nuts and seeds were used to estimate refined grains. Sodium used fruits, grains, and water in its scoring rules. Saturated fats used sugar-sweetened beverages, grains, and nuts and seeds in its scoring rules.

### 3.2. Food Group Intake Correlation

The DSQ measured a different set of food groups. To determine how the sHEI measures those food groups in comparison to the DSQ, we also trained CRT models using the DSQ food group intake as the dependent variable (i.e., ground truth) whereas the sHEI questions as the predictors. Pearson’s correlation in the DSQ items between the sHEI and the actual DSQ instrument from the training data (*n* = 50) can be found in Table 3, Column (a). These results showed that these food group intake amounts estimated using the sHEI had a moderate to high correlation with the DSQ.

Finally, the correlation between the carotenoid measurements and the sHEI scores showed that the sHEI’s estimated green vegetables intake and dermal carotenoid levels had a moderate correlation of 0.45, and the sHEI’s the total Healthy Eating Index and the dermal carotenoid levels had a moderate correlation of 0.44.

### 3.3. Confirmatory Analysis

A sample of *n* = 505 eligible participants took an online survey that included the sHEI, the DSQ, and demographics. Records (*n* = 107) were dropped due to missing values for at least one sHEI question. This left a sample of *n* = 398 responses for analysis. Among these subjects, 299 (75%) were female, and participant ages ranged from 18 to 40, with a mean of 20 and a median of 19. BMI ranged from 13.6 to 43.6 kg/m^2^ (with mean 23.7 and standard deviation 4.6).

Because the confirmatory sample did not complete 24 h recalls to compare the reported sHEI answers, the DSQ was used as a comparison tool. The correlations in food consumption items between sHEI estimates (as trained from the 24 h recall data using the original training sample) and the DSQ estimates of the confirmatory sample showed a moderate to strong correlation for all items (Table 3, Column b).

### 3.4. Overall Tool

Out of 35 questions developed for the sHEI, 7 questions that were originally included for use if a respondent reported consuming a food less than once a day were removed during analysis because they were found not to contribute to the overall sHEI DQ score or needed to estimate any specific food group. These seven questions (about less than daily consumption patterns) that were removed were for fruit, 100% juice, vegetables, green vegetables, beans, and nuts/seeds. Another two questions were also removed, one about oil, and another about salt, because they did not contribute to the sHEI DQ score or any food group estimation. Finally, questions were removed about meat and red/orange vegetables. These removed meat and red/orange vegetable questions represented four question in total (each having a question on total intake and a follow-up question if less than daily consumption reported). The final scoring system for the sHEI DQ score was generated using 17 of the sHEI questions and one question on gender for a total of 18 items (Appendix B). The remaining questions were retained in the sHEI for estimating intake of specific food groups (Appendix C). Overall a 22-item tool remained that could be used to calculate a sHEI score and estimate individual’s food component consumption (Appendix A).

## 4. Discussion

The sHEI tool has been developed and validated and can be used to assess an adult’s DQ in an online or paper survey administration process. Using multiple independent methods to develop a survey tool strengthens the instrument’s validity [43]. The use of convergent validity to assess the relationship between the newly developed sHEI tool to other measures of diet quality, independently, is a strong way to approach validation [44]. The subjects’ dietary intake, although slightly different among the NDSR, the DSQ, and sHEI tools (Table 1), overall was found to be similar, indicating overall consistency among the NDSR, the DSQ, and sHEI tools. The sHEI instrument was correlated with the HEI scoring system using 24 h recalls, the DSQ, and dermal carotenoid levels. The comparison of the sHEI instrument and the other instruments indicates this tool can adequately provide information for some individual nutrient and food group consumption amounts (fruits, vegetables, dairy, added sugar, sugar from sugar-sweetened beverages, and calcium) and a total diet quality score that is well correlated to other well-developed and validated instruments/approaches. The final 22-item tool has less respondent and researcher burden than many other dietary quality assessment instruments currently available in dietary research.

The use of CRT is both a strength and weakness of this work. CRT creates an algorithm in which predictor variables are identified from a larger pool of potential variables that predict the target variable [47]. It is a decision tree where each fork is a split in a predictor variable (e.g., different potential answers to a survey question) and each node at the end has a prediction for the target variable. One of the potential problems with CRT is that the same items, or a combination of those items, are used to calculate more than one component score. This could be considered “double dipping” and could result in inflation of the overall correlation. Another potential problem with the CRT is that it could “pull” a factor that an expert would not see as logically related. It may appear to be a statistical artifact instead of an actual predictive variable of interest. Because of this, the factors that CRT identifies as useful in an algorithm should be reviewed by experts to verify the practical use. In this work, all CRT branches and variables were reviewed by experts before incorporation in the final scoring system.

Another strength of the development and validation process used with the sHEI was the systematic approach used to create the sHEI scoring process. The process used provided both clinically relevant and data-driven approaches, allowing the instrument to more accurately estimate food group consumption and total DQ score. The data-driven approach provided scoring rules that experts used in an interactive process to inform the data-driven approach. If either one (expert- or data-driven) approach had been used by itself, the accuracy of the scoring process would not have been as strong.

As with any self-reported diet assessment, there remains many limitations because of response bias, participants’ recall limitations, and participants’ knowledge of foods and serving sizes [12]. For example, it became apparent to researchers in this study that participants could not adequately report total grains and whole grains. Frolich and Aman argue that inconsistent definitions of whole grains leaves consumers confused about what a whole grain is and thus, inaccurate reporting of whole grains is speculated [49]. In this study, it appeared that individuals may not have understood the difference between the total grains and whole grains as they often reported the same intake of whole grains as they did for their total grains. This discrepancy prevented the researchers from being able to calculate refined grains and provide a higher correlated score for this food group. Similarly, with the saturated fat variable, it appeared that people were not very good at identifying how much saturated fat they consumed. Respondents were given the options of almost none/never, some, or a lot and many people who said “almost none/never” actually consumed about the same amount as those who said they consumed some or a lot. Thus, the scores for the sHEI created by the content experts, 10 for none/never, 5 for some, and 0 for a lot were adjusted to a 7, 5, and 3, respectively, to account for the over and under reporting observed in this food group area in the data-driven scoring determination process.

Sample size was another limitation of this work. The initial sample used to validate the sHEI with the 24 h recall was smaller than other diet assessment validation studies have previously used. However, this study did use a larger confirmatory sample to validate the questionnaire and its scoring system. The correlations found in the confirmatory sample, though consistently lower than the initial sample, provided another comparison for the sHEI. Although the correlations may be lower when this survey tool is used with a larger sample, the correlations in this confirmatory work were still at acceptable levels [48], and the practical benefits of not having to do repeated 24 h recalls with a large number of participants may make these reductions in correlations acceptable. The sample size also posed issues for the development of the scoring system as the NDSR data (produced from the repeated 24 h recalls) could not be used as the only validation comparison since with the small sample, a single or a few individuals could disproportionately affect the NDSR-generated HEI score. This potential limitation was addressed by having multiple tools used in the validation process.

Another potential limitation of comparing an overall DQ score with 24 h recalls is that the sHEI asked for typical average behavior, whereas in the 24 h recalls, dietary behaviors observed may be unique to that time point. For example, if someone consumed a specific food group on a specific day documented in the 24 h recall, it might have looked as if they ate a lot of that food; whereas, in the sHEI, they may actually report more accurately that the food was consumed only once a month. Although this is a limitation, if biomarker data are unavailable, 24 h recalls are considered the “gold standard” for dietary tool validation work [12]. Again, this limitation was addressed by using multiple tools in the validation process with the confirmatory analysis using the sHEI and the DSQ with a larger sample size. This confirmatory process used two tools that both relied on average reported intake and indicated that all items had a moderate to strong correlation. Although using a confirmatory process was a strength in this work, the confirmatory sample correlations were systematically lower, which could have been explained by context shift (e.g., subjects’ level of commitment). The most parsimonious reason would be that the algorithm was developed with one sample which maximized the fit and it would be expected to be lower with a different sample. This context shift can happen when a new sample has differences in factors such as demographics, socioeconomic, and behavior variables.

## 5. Conclusions

Despite these limitations, a systematically developed, validated, brief tool was produced from this work. This 22-item tool is free for use, has low respondent and researcher burden, and can be used to accurately estimate overall DQ and some individual nutrient and food group consumption (fruits, vegetables, dairy, added sugar, sugar from sugar-sweetened beverages, and calcium). The accuracy of this tool in estimating intake of these specific food components may make this tool especially useful in research and health promotion program evaluations as these are areas of intake associated with disease prevention. This practical application to health-related investigations, combined with the low respondent burden, makes this tool especially positioned to be useful in a variety of settings. The sHEI assessment tool would benefit from further validation in a variety of populations, such as those who are older or younger than college age students and those with specific eating patterns (vegetarian, vegan, “fad” diet, or disease-specific diet pattern such as gluten-free).

## Figures and Tables

**Table 1:** Estimated Food Group Consumption by Instrument (24 h Recall, the sHEI, and the DSQ).

Dietary Factor and Instrument	24 h Recall		sHEI		DSQ	
	Mean	±sd	Mean	±sd	Mean	±sd
Total vegetable servings in cup equivalents (including legumes and French fries)	1.37	±0.83	1.31	±0.59	1.55	±0.35
Total vegetable servings in cup equivalents including legumes and excluding French fries	1.12	±0.82	1.04	±0.58	1.42	±0.37
Greens and beans servings in cup equivalents	0.28	±0.31	0.22	±0.15	N/A	N/A
Total fruit servings in cup equivalents	0.68	±0.72	0.57	±0.49	1.06	±0.45
Total fruit and vegetable servings in cup equivalents	2.05	±1.33	1.92	±0.67	2.59	±0.74
Total fruit and vegetable servings in cup equivalents including legumes and excluding French fries	1.80	±1.34	1.66	±0.77	2.50	±0.73
Whole fruit servings in cup equivalents	0.59	±0.63	0.48	±0.37	N/A	N/A
Dairy servings in cup equivalents	1.61	±2.18	1.35	±0.67	1.79	±0.73
Whole grain servings in ounce equivalents	1.46	±1.41	1.23	±0.77	0.80	±0.31
Total protein servings in ounce equivalents	6.77	±2.79	6.60	±1.67	N/A	N/A
Seafood and plant protein servings in ounce equivalents	1.37	±1.51	1.09	±0.94	N/A	N/A
Refined grains in ounce equivalents	6.01	±6.59	5.39	±2.86	N/A	N/A
Sodium (g)	3.39	±1.88	3.30	±1.11	N/A	N/A
Added sugars in tsp equivalents ^1^	14.10	±9.32	13.28	±5.53	17.15	±8.18
Added sugars from sugar-sweetened beverages in tsp equivalents ^2^	5.81	±7.95	4.49	±4.70	7.90	±7.02
Fiber (g)	18.02	±9.33	17.40	±5.32	16.68	±3.48
Calcium (mg)	975.59	±657.74	936.20	±404.77	1049.21	±242.11

The NDSR: the Nutrition Data System for Research; the sHEI: the Short Healthy Eating Index; the DSQ: the Dietary Screener Questionnaire; 24 h recall: 24 h recall; g: grams; mg: milligrams. ^1^ The NDSR provided an estimate of added sugars by the unit of total calories (kcal). They were converted into tsp equivalents in this table assuming every 16 kcals converts to 1 tsp. ^2^ The NDSR only provides an estimate of sweetened beverage servings. We estimate the added sugar content in those beverages using sweetened beverage servings times 6.5.

**Table 2:** The Pearson’s Correlation between the sHEI and the 24 h Recall NDSR in Estimated HEI Component and Total Scores.

HEI Component	Correlation
(1) Total Fruits	0.64
(2) Whole Fruits	0.57
(3) Total Vegetables	0.53
(4) Greens and Beans	0.49
(5) Whole Grains	0.62
(6) Dairy	0.48
(7) Total Protein Foods	0.51
(8) Seafood and Plant Proteins	0.56
(9) Fatty Acids	0.44
(10) Refined Grains	0.62
(11) Sodium	0.58
(12) Added Sugars	0.47
(13) Saturated Fats	0.51
Total Score	0.79

The sHEI: the Short Healthy Eating Index; 24 h recall: 24 h recall; the NDSR: the Nutrition Data System for Research; the HEI: the Healthy Eating Index; CRT: classification and regression tree.

**Table 3:** The Pearson’s Correlation between the sHEI and the DSQ.

Dietary Factor from the DSQ (Predicted Intake per Day)	(a) Training Sample (*n* = 50)	(b) Confirmatory Sample (*n* = 398)
Total fruit and vegetable servings in cup equivalents including legumes and French fries (DSQfvl)	0.74	0.49
Total fruit and vegetable servings in cup equivalents including legumes and excluding French fries (DSQfvlnf)	0.73	0.49
Total fruit servings in cup equivalents (DSQfrt)	0.85	0.49
Total vegetable servings in cup equivalents including legumes and French fries (DSQvlall)	0.63	0.60
Total vegetable servings in cup equivalents including legumes and excluding French fries (DSQvlnf)	0.63	0.59
Dairy servings in cup equivalents (DSQdairy)	0.69	0.49
Added sugars in tsp (DSQsug)	0.70	0.49
Added sugars from sugar-sweetened beverages in tsp equivalents (DSQssb)	0.74	0.57
Whole grains in ounce equivalents (DSQwhgr)	0.65	0.29
Fiber in grams (DSQfib)	0.67	0.44
Calcium in milligrams (DSQcalc)	0.78	0.66

The sHEI: the Short Healthy Eating Index; the DSQ: the Dietary Screener Questionnaire. Note: the DSQ variable names are indicated in parentheses in column 1.

## Appendix A. The sHEI Survey (The Original Survey Has Pictures with Each Question. That Version Is Available upon Request with the Contact Author.)

### Q1 (Fruit)

On average, how many servings of fruit (not including juice) do eat per day?

Example: 1 serving fruit = 1/2 cup cut-up fruit, 1/2 a banana, or one small piece of whole fruit (apple, orange, pear, etc.) One small piece of whole fruit is the size of a baseball. 1/2 cup cut-up fruit is the size of a computer mouse.

(1) Less than 1

(2) 1

(3) 2

(4) 3

(5) 4

(6) 5

(7) 6 or more

(8) Choose not to answer

### Q2 (Fruitjuice)

On average, how many servings of 100% fruit juice do you drink per day?

NOTE: Do not include fruit flavored drinks such as Hi-C, Tang, Sunny-D, etc.

Example: 1 serving juice = 1/2 cup 100% fruit juice (apple, grape, orange, etc.), 1 cup of juice = juice box.

(1) Less than 1

(2) 1

(3) 2

(4) 3

(5) 4

(6) 5

(7) 6 or more

(8) Choose not to answer

### Q3 (Vege)

Now, think about all of the vegetables you eat in a day.

On average, how many servings of vegetables do you eat per day?

NOTE: Any vegetable or 100% vegetable juice counts as a member of the vegetable group.

Example: 1 serving = 1 cup raw vegetables, 1 cup of salad, 1/2 cup cooked vegetables, or 1/2 cup 100% vegetable juice. One cup raw vegetables is the size of a baseball. 1/2 cup cooked vegetables is the size of a computer mouse.

(1) Less than 1

(2) 1

(3) 2

(4) 3

(5) 4

(6) 5

(7) 6 or more

(8) Choose not to answer

### Q4 (Greenvege)

Now, think about just the green vegetables you eat in a day like spinach, green beans, kale, broccoli, zucchini, or other mostly green vegetables.

On average, how many servings of green vegetables do you eat per day?

NOTE: Do not include starchy vegetables like green peas.

Example: 1 serving = 1 cup raw vegetables or 1/2 cup cooked vegetables. 1 cup raw vegetables is the size of a baseball. 1/2 cup cooked vegetables is the size of a computer mouse.

(1) Less than 1

(2) 1

(3) 2

(4) 3

(5) 4

(6) 5

(7) 6 or more

(8) Choose not to answer

### Q5 (Starchy)

Now, think about just the starchy vegetables you eat in a day like corn, green peas, or potatoes.

On average, how many servings of starchy vegetables do you eat per day?

Examples: 1 serving = 1 cup raw vegetable or 1/2 cup cook vegetables. 1 cup raw vegetables is the size of a computer mouse.

(1) Less than 1

(2) 1

(3) 2

(4) 3

(5) 4

(6) 5

(7) 6 or more

(8) Choose not to answer

### Q6 (Grains)

On average, how many servings of grains do you eat per day?

Examples: 1 serving = 1 slice of bread; 1/2 cup grits, 1 cup of ready-to-eat cereal, 1/2 cup oatmeal, 1 small tortilla, 1/2 cup cooked rice, or 1/2 cup pasta. 1 cup ready-to-eat cereal is the size of a baseball.

(1) Less than 1

(2) 1

(3) 2

(4) 3

(5) 4

(6) 5

(7) 6 or more

(8) Choose not to answer

### Q7 (Grains2) If “Less Than 1” is Selected for Q6

On average, how often do you eat grains?

Examples: 1 serving = 1 slice of bread; 1/2 cup grits, 1 cup of ready-to-eat cereal, 1/2 cup oatmeal, 1 small tortilla, 1/2 cup cooked rice, or 1/2 cup pasta.

(1) A couple times per week

(2) A couple times per month

(3) A couple times per year

(4) Almost never

(5) Never

(6) Choose not to answer

### Q8 (Wholegrains)

Now, just think about whole grains you eat like whole wheat bread, whole grain crackers, brown rice, or oatmeal.

On average, how many servings of whole grains do you eat per day?

Examples: 1 serving = 1 slice whole wheat bread, 5–6 whole grain crackers, 3 cups popcorn, 1/2 cup cooked brown rice, or 1/2 cup oatmeal.

(1) Less than 1

(2) 1

(3) 2

(4) 3

(5) 4

(6) 5

(7) 6 or more

(8) Choose not to answer

### Q9 (Wholegrains2) If “Less Than 1” is Selected for Q8

On average, how often do you eat whole grains?

Examples: 1 serving = 1 slice whole wheat bread, 5–6 whole grain crackers, 3 cups popcorn, 1/2 cup cooked brown rice, or 1/2 cup oatmeal.

(1) A couple times per week

(2) A couple times per month

(3) A couple times per year

(4) Almost never

(5) Never

(6) Choose not to answer

### Q10 (Milk)

On average, how many servings of milk do you eat or drink per day?

Examples: 1 serving = 1 cup of milk, 1 cup of yogurt, 1.5 ounces of natural cheese, or 2 ounces of processed cheese. 1 cup of milk is the size of a carton of milk. 1 serving of cheese is the size of your index finger.

(1) Less than 1

(2) 1

(3) 2

(4) 3

(5) 4

(6) 5

(7) 6 or more

(8) Choose not to answer

### Q11 (Milk2) If “Less Than 1” is Selected for Q10

On average, how often do you drink or eat milk products?

Examples: 1 serving = 1 cup of milk, 1 cup of yogurt, 1.5 ounces of natural cheese, or 2 ounces of processed cheese.

(1) A couple times per week

(2) A couple times per month

(3) A couple times per year

(4) Almost Never

(5) Never

(6) Choose not to answer

### Q12 (Lowfatmilk)

Now, just think about the milk products you eat per day.

On average, how many servings of low-fat milk products do you eat per day?

Examples: 1 serving = 1 cup of skim milk, 1 cup of low fat yogurt, or 1.5 ounces of low-fat cheese. 1 cup of milk is the size of a milk carton. 1 serving of cheese is the size of your index finger.

(1) Less than 1

(2) 1

(3) 2

(4) 3

(5) 4

(6) 5

(7) 6 or more

(8) Choose not to answer

### Q13 (Lowfatmilk2) If “Less Than 1” is Selected for Q12

On average, how often do you drink or eat low-fat milk products?

Examples: 1 serving = 1 cup of skim milk, 1 cup of low fat yogurt, or 1.5 ounces of low-fat cheese.

(1) A couple times per week

(2) A couple times per month

(3) A couple times per year

(4) Almost never

(5) Never

(6) Choose not to answer

### Q14 (Beans)

On average, how many servings of beans (legumes) do you eat per day?

NOTE: all foods made from dry beans, canned beans, peas, and lentils are considered part of this group.

Examples: 1 serving= 1/2 cup cooked beans. 1/2 cup cooked beans is the size of a computer mouse.

(1) Less than 1

(2) 1

(3) 2

(4) 3

(5) 4

(6) 5

(7) 6 or more

(8) Choose not to answer

### Q15 (Nutseeds)

On average, how many servings of nuts or seeds do you eat per day?

NOTE: 1 serving = 1 tbsp of peanut butter; 1/2 ounces of nuts or seeds. 1 tbsp of peanut butter is the size of the tip of your thumb.

(1) Less than 1

(2) 1

(3) 2

(4) 3

(5) 4

(6) 5

(7) 6 or more

(8) Choose not to answer

### Q16 (Seafood)

On average, how many servings of seafood do you eat per day?

NOTE: All foods made of fish, shrimp, crab, and shellfish are considered part of this group.

Examples: 1 serving = 3 ounces of fish. 3 ounces of fish is the size of a deck of cards.

(1) Less than 1

(2) 1

(3) 2

(4) 3

(5) 4

(6) 5

(7) 6 or more

(8) Choose not to answer

### Q17 (Seafood2) If “Less Than 1” is Selected for Q16

On average, how often do you eat seafood?

NOTE: All foods made of fish, shrimp, crab, and shellfish are considered part of this group.

Examples: 1 serving = 3 ounce of fish.

(1) A couple times per week

(2) A couple times per month

(3) A couple times per year

(4) Almost never

(5) Never

(6) Choose not to answer

### Q18 (Ssb)

On average, how many sugar-sweetened beverages do you drink per day?

Examples: 12 oz of soft drinks/soda, fruit flavored drinks, sweetened coffee, and sweet tea. Do not include milk or 100% fruit juice. 12 oz of soda is the size of one can.

(1) Less than 1

(2) 1

(3) 2

(4) 3

(5) 4

(6) 5

(7) 6 or more

(8) Choose not to answer

### Q19 (Ssb2) If “Less Than 1” is Selected for Q18

On average, how often do you drink sugar-sweetened beverages?

Examples: 12 oz of soft drinks/soda, fruit flavored drinks, sweetened coffee, and sweet tea. Do not include milk or 100% fruit juice.

(1) A couple times per week

(2) A couple times per month

(3) A couple times per year

(4) Almost never

(5) Never

(6) Choose not to answer

### Q20 (Addedsugars)

On average, how much added sugars do you consume per day?

NOTE: Added sugars are often in foods such as breads, cakes, candy, sweet tea, jam, ice cream, or sugar added to food at the table. Do not include naturally occurring sugars such as lactose in milk or fructose in fruits.

Examples: white sugar, brown sugar, raw sugar, corn syrup, corn-syrup solids, high-fructose corn syrup, malt syrup, maple syrup, pancake syrup, fructose sweetener, liquid fructose, honey, molasses, and dextrose.

(1) None/almost none

(2) Some

(3) A lot

(4) Choose not to answer

### Q21 (Satfat)

How many servings of saturated fat do you consume on average per day?

NOTE: Saturated fats for these purposes should be considered to be solid fats. Solid fats are fats that are solid at room temperature.

Examples: butter, cakes, cookies, Crisco, coconut oil, beef fat (tallow, suet), chicken fat (lard), stick margarine, and shortening.

(1) None/almost none

(2) Some

(3) A lot

(4) Choose not to answer

### Q22 (Water)

On average, how much water do you drink per day?

(1) None/almost none

(2) Some

(3) A lot

(4) Choose not to answer

## Appendix B. sHEI Scoring Instructions for Estimating sHEI Total Dietary Quality Score

To score the HEI component scores and the total, we need the following 17 items: Q1 (fruit), Q2 (fruitjuice), Q4 (greenvege), Q5 (startchy), Q6 (grains), Q8 (wholegrains), Q10 (milk), Q11 (milk2), Q12 (lowfatmilk), Q13 (lowfatmilk2), Q14 (beans), Q15 (nutseeds), Q16 (seafood), Q17 (seafood2), Q18 (ssb), Q20 (addedsugars), Q21 (satfat), Q22 (water), and Gender.

*Total Fruits Component (total_fruits), 0–5*
IF Q1 (fruit) = 1 THEN total_fruits_Q1 = 0; IF Q1 (fruit) = 2 THEN total_fruits_Q1 = 2; IF Q1 (fruit) = 3 THEN total_fruits_Q1 = 3.5; IF Q1 (fruit) = 4,5,6,7 THEN total_fruits_Q1 = 5. IF Q2 (fruitjuice) = 1 THEN total_fruits_Q2 = 0; IF Q2 (fruitjuice) = 2 THEN total_fruits_Q2 = 2; IF Q2 (fruitjuice) = 3 THEN total_fruits_Q2 = 3.5; IF Q2 (fruitjuice) = 4,5,6,7 THEN total_fruits_Q2 = 5. total_fruits = total_fruits_Q1 + total_fruits_Q2. IF total_fruits > 5 THEN total_fruits = 5.
*Whole Fruits Component (whole_fruits), 0–5*
IF Q1 (fruit) = 1 THEN whole_fruits = 0; IF Q1 (fruit) = 2 THEN whole_fruits = 2.5; IF Q1 (fruit) = 3,4,5,6,7 THEN whole_fruits = 5.
*Total Vegetables Component (tot_veg), 0–5*
IF Q4 (greenvege) = 1 THEN tot_veg = 1.60; IF Q5 (startchy) = 2,3,4,5,6,7 AND Q4 (greenvege) = 2 THEN tot_veg = 2.46; IF Q5 (startchy) = 2,3,4,5,6,7 AND Q4 (greenvege) = 3,4,5,6,7 THEN tot_veg = 3.24; IF Q5 (startchy) = 1 AND Q4 (greenvege) = 2,3,4,5,6,7 THEN tot_veg = 3.56.
*Greens and Beans Component (greens_beans), 0–5*
IF Q4 (greenvege) = 1 THEN greens_beans_Q7 = 0; IF Q4 (greenvege) = 2,3,4,5,6,7 THEN greens_beans_Q7 = 5. IF Q14 (beans) = 1 THEN greens_beans_Q14 = 0; IF Q14 (beans) = 2,3,4,5,6,7 THEN greens_beans_Q14 = 5. greens_beans = greens_beans_Q4 + greens_beans_Q14. IF greens_beans > 5 THEN greens_beans = 5.
*Whole Grains Component (whole_grains), 0–10*
IF Q8 (wholegrains) = 1 THEN whole_grains = 0.51; IF Gender = M AND Q8 (wholegrains) = 2,3,4,5,6,7 THEN whole_grains = 2.97; IF Gender = F AND Q8 (wholegrains) = 2,3 THEN whole_grains = 5.20; IF Gender = F AND Q8 (wholegrains) = 4,5,6,7 THEN whole_grains = 6.94.
*Dairy Component (Dairy), 0–10*
IF Gender = M AND Q10 (milk) = 1,2,3 THEN dairy = 3.22; IF Gender = F AND Q10 (milk) = 1,2,3 AND Q12 (lowfatmilk) = 1 THEN dairy = 3.32; IF Gender = F AND Q10 (milk) = 1,2,3 AND Q12 (lowfatmilk) = 2,3,4,5,6,7 THEN dairy = 4.81; IF Q10 (milk) = 4,5,6,7 THEN dairy = 6.51.
*Total Protein Foods Component (tot_proteins), 0–5*
IF Gender = M AND Q16_17 (seafood_combo) = 1,2,3,4 THEN tot_proteins = 4.11; IF Gender = M AND Q16_17 (seafood_combo) = 5,6,7,8,9,10,11 THEN tot_proteins = 4.98; IF Gender = F THEN tot_proteins = 4.97.
*Seafood and Plant Protein Component (Seafood_Plant), 0–5*
IF Gender = M AND Q15 (nutseeds) = 1,2 THEN seafood_plant = 0.49; IF Gender = F AND Q15 (nutseeds) = 1,2 THEN seafood_plant = 1.50; IF Q15 (nutseeds) = 3,4,5,6,7 THEN seafood_plant = 4.20.
*Fatty Acid Ratio Component (Fatty_Acid), 0–10*
IF Q10 (milk) = 4,5,6,7 THEN fatty_acid = 2.56; IF Q21 (satfat) = 2,3 AND Q10_11 (milk_combo) = 1,2,3,4,5,6,7 AND Q12–13 (lowfatmilk_combo) = 1,2 THEN fatty_acid = 2.63; IF Q21 (satfat) = 2,3 AND Q10_11 (milk_combo) = 1,2,3,4,5,6,7 AND Q12_13 (lowfatmilk_combo) = 3,4,5,6,7,8,9,10,11 THEN fatty_acid = 4.54; IF Q21 (satfat) = 1 AND Q10_11 (milk_combo) = 1,2,3,4,5,6,7 THEN fatty_acid = 5.93.
*Refined Grains Component (Refined_Grains), 0–10*
IF Q4 (greenvege) = 1 THEN refined_grains = 2.13; IF Q6 (grains) = 3,4,5,6,7 AND Q16 (seafood) = 2,3,4,5,6,7 AND Q4 (greenvege) = 2,3,4,5,6,7 THEN refined_grains = 2.27; IF Q6 (grains) = 3,4,5,6,7 AND Q15 (nutseeds) = 1,2 AND Q16 (seafood) = 1 AND Q4 (greenvege) = 2,3,4,5,6,7 THEN refined_grains = 4.73; IF Q6 (grains) = 3,4,5,6,7 AND Q15 (nutseeds) = 3,4,5,6,7 AND Q16 (seafood) = 1 AND Q4 (greenvege) = 2,3,4,5,6,7 THEN refined_grains = 8.45; IF Q6 (grains) = 1,2 AND Q4 (greenvege) = 2,3,4,5,6,7 THEN refined_grains = 9.25.
*Sodium Component (Sodium), 0–10*
IF Q1 (fruit) = 1,2 AND Q6 (grains) = 3,4,5,6,7 AND Q22 (water) = 3 THEN sodium = 0.70; IF Q1 (fruit) = 3,4,5,6,7 AND Q6 (grains) = 3,4,5,6,7 AND Q22 (water) = 3 THEN sodium = 2.30; IF Q6 (grains) = 3,4,5,6,7 AND Q22 (water) = 1,2 THEN sodium = 4.94; IF Q6 (grains) = 1,2 THEN sodium = 6.07.
*Added Sugars Component (Added_Sugars), 0–10*
IF Q18 (ssb) = 1 THEN sugar_calories_Q18 = 0; IF Q18 (ssb) = 2 THEN sugar_calories_Q18 = 156; IF Q18 (ssb) = 3 THEN sugar_calories_Q18 = 312; IF Q18 (ssb) = 4 THEN sugar_calories_Q18 = 468; IF Q18 (ssb) = 5 THEN sugar_calories_Q18 = 624; IF Q18 (ssb) = 6 THEN sugar_calories_Q18 = 780; IF Q18 (ssb) = 7 THEN sugar_calories_Q18 = 936. IF Q20 (addedsugars) = 1 THEN sugar_calories_Q20 = 130; IF Q20 (addedsugars) = 2 THEN sugar_calories_Q20 = 260; IF Q20 (addedsugars) = 3 THEN sugar_calories_Q20 = 520. sugar_calories = sugar_calories_Q18 + sugar_calories_Q20. IF sugar_calories < =130 THEN added_sugars = 10; IF sugar_calories > 130 AND sugar_calories < 520 THEN added_sugars = 5; IF sugar_calories > =520 THEN added_sugars = 0.
*Saturated Fats Component (sat_fat), 0–10*
IF Q18 (ssb) = 3,4,5,6,7 THEN sat_fat = 1.82; IF Q6 (grains) = 1,2 AND Q18 (ssb) = 1,2 THEN sat_fat = 3.20; IF Q6 (grains) = 3,4,5,6,7 AND Q15 (nutseeds) = 1,2 AND Q18 (ssb) = 1,2 THEN sat_fat = 4.64; IF Q6 (grains) = 3,4,5,6,7 AND Q15 (nutseeds) = 3,4,5,6,7 AND Q18 (ssb) = 1,2 THEN sat_fat = 6.56.
*Total DQ Score (0–100)*
tot_score = total_fruits + whole_fruits + tot_veg + greens_beans + whole_grains + dairy + tot_proteins + seafood_plant + fatty_acid + refined_grains + sodium + added_sugars + sat_fat

## Appendix C. sHEI Scoring Instructions for Estimating Food Group Consumption

To score the following food group intake amounts, we need the following 10 items: Q1 (fruit), Q3 (vege), Q4 (greenvege), Q6 (grains), Q7 (grains2), Q8 (wholegrains), Q9 (wholegrains2), Q10 (milk), Q18 (ssb), Q19 (ssb2), and Gender.

### Total Fruit and Vegetable Servings in Cup Equivalents Including Legumes and French Fries (DSQfvl)

IF Q1 (fruit) = 1 THEN DSQfvl = 1.81;

IF Gender = F AND Q1 (fruit) = 2,3 THEN DSQfvl = 2.22;

IF Gender = M AND Q1 (fruit) = 2,3 THEN DSQfvl = 2.53;

IF Q1 (fruit) = 4,5,6,7 THEN DSQfvl = 3.21.

### Total Fruit and Vegetable Servings in Cup Equivalents Including Legumes and Excluding French Fries (Dsqfvlnf)

IF Q1 (fruit) = 1 THEN DSQfvlnf = 1.72;

IF Gender = F AND Q1 (fruit) = 2,3 THEN DSQfvlnf = 2.15;

IF Gender = M AND Q1 (fruit) = 2,3 THEN DSQfvlnf = 2.43;

IF Q1 (fruit) = 4,5,6,7 THEN DSQfvlnf = 3.10.

### Total Fruit Servings in Cup Equivalents (Dsqfrt)

IF Q1 (fruit) = 1 THEN DSQfrt = 0.52;

IF Q1 (fruit) = 2 THEN DSQfrt = 0.78;

IF Q1 (fruit) = 3 THEN DSQfrt = 0.99;

IF Q1 (fruit) = 4,5,6,7 THEN DSQfrt = 1.52.

### Total Vegetable Servings in Cup Equivalents Including Legumes and French Fries (Dsqvlall)

IF Q3 (vege) = 1,2 THEN DSQvlall = 1.34;

IF Gender = F AND Q3 (vege) = 3,4 THEN DSQvlall = 1.40;

IF Gender = F AND Q3 (vege) = 5,6,7 THEN DSQvlall = 1.68;

IF Gender = M AND Q3 (vege) = 3,4,5,6,7 THEN DSQvlall = 1.84.

### Total Vegetable Servings in Cup Equivalents Including Legumes and Excluding French Fries (Dsqvlnf)

IF Q3 (vege) = 1,2 THEN DSQvlnf = 1.19;

IF Gender = F AND Q3 (vege) = 3,4 THEN DSQvlnf = 1.28;

IF Gender = F AND Q3 (vege) = 5,6,7 THEN DSQvlnf = 1.59;

IF Gender = M AND Q3 (vege) = 3,4,5,6,7 THEN DSQvlnf = 1.71.

### Dairy Servings in Cup Equivalents (Dsqdairy)

IF Q10 (milk) = 1 THEN DSQdairy = 1.16;

IF Gender = F AND Q10 (milk) = 2,3 THEN DSQdairy = 1.48;

IF Gender = M AND Q10 (milk) = 2,3 THEN DSQdairy = 1.90;

IF Q10 (milk) = 4,5,6,7 THEN DSQdairy = 2.36.

### Added Sugars in Teaspoon Equivalents (Dsqsug)

IF Q18_19 (ssb_combo) = 1,2,3,4 THEN DSQsug = 13.26;

IF Q18_19 (ssb_combo) = 5,6 THEN DSQsug = 16.00;

IF Q18 (ssb) = 3,4,5,6,7 THEN DSQsug = 26.87.

### Added Sugars from Sugar-Sweetened Beverages in Teaspoon Equivalents (DSQssb)

IF Gender = F AND Q18_19 (ssb_combo) = 1,2,3,4 THEN DSQssb = 4.13;

IF Gender = M AND Q18_19 (ssb_combo) = 1,2,3,4 THEN DSQssb = 5.73;

IF Q18_19 (ssb_combo) = 5,6 THEN DSQssb = 6.79;

IF Q18 (ssb) = 3,4,5,6,7 THEN DSQssb = 15.78.

### Whole Grains in Ounce Equivalents (DSQwhgr)

IF Q8_9 (wholegrains_combo) = 1,2,3,4 THEN DSQwhgr= 0.50;

IF Q6_7 (grains_combo) = 1,2,3,4,5,6 AND Q8_9 (wholegrains_combo) = 5,6,7

THEN DSQwhgr = 0.63;

IF Q6 (grains) = 3,4,5,6,7 AND Q8_9 (wholegrains_combo) = 5,6,7

THEN DSQwhgr = 0.77;

IF Q8 (wholegrains) = 4,5,6,7 THEN DSQwhgr = 1.01.

### Fiber in Grams (DSQfib)

IF Gender = F AND Q1 (fruit) = 1,2,3 THEN DSQfib = 13.69;

IF Gender = M AND Q1 (fruit) = 1,2,3 THEN DSQfib = 16.74;

IF Q1 (fruit) = 4,5,6,7 THEN DSQfib = 19.32.

### Calcium in Milligrams (DSQcalc)

IF Gender = F AND Q10 (milk) = 1,2 THEN DSQcalc = 851.61;

IF Gender = F AND Q10 (milk) = 3,4,5,6,7 THEN DSQcalc = 1010.78;

IF Gender = M AND Q10 (milk) = 1,2 THEN DSQcalc = 1062.55;

IF Gender = M AND Q10 (milk) = 3,4,5,6,7 THEN DSQcalc = 1319.71.

### Green Vegetables in Cup Equivalents

IF Q4 (greenvege) = 1 THEN greens = 0.00

IF Q3 (vege) = 1,2,3,4 AND Q7 (greenvege) = 2,3,4,5,6,7 THEN greens = 0.13

IF Q3 (vege) = 5,6,7 AND Q7 (greenvege) = 2,3,4,5,6,7 THEN greens = 0.31
